# Choosing wisely: comparing the carbon footprint of three respiratory sampling techniques for ventilator-associated pneumonia

**DOI:** 10.1186/s13613-025-01597-y

**Published:** 2025-12-08

**Authors:** Adam Celier, Charlotte Correard, Ines de Maisoncelle, Eric Dupont, Alexandre Demoule, Marie Lecronier

**Affiliations:** 1 Service de Médecine Intensive et Réanimation (Département R3S), AP-HP, Groupe Hospitalier Universitaire APHP-Sorbonne Université, site Pitié-Salpêtrière, Paris, F-75013 France; 2 Direction de la Stratégie et de la Transformation, AP-HP, Paris, France; 3 UMRS1158 Neurophysiologie Respiratoire Expérimentale et Clinique, Sorbonne Université, INSERM, Paris, 75005 France

**Keywords:** Intensive care, Ventilator-associated pneumonia, Carbon footprint, Sustainable healthcare

## Abstract

**Background:**

Intensive care units (ICU) play a significant role in healthcare global greenhouse gas emissions. Ventilator-associated pneumonia (VAP) is a common ICU-acquired infection, and while microbiological confirmation is essential, the optimal sampling method remains controversial. This study compares the carbon footprint of three diagnostic techniques for VAP—tracheal aspiration (TA), blind bronchial sampling (BBS) and bronchoalveolar lavage (BAL) using single-use bronchoscopes—while also assessing their economic cost and professional impact to support more sustainable decision-making in the ICU.

**Methods:**

The carbon footprint of each technique was estimated using a simplified Life Cycle Assessment (LCA) methodology via the “Carebone©” tool. Emission factors for drugs and devices were calculated. The economic costs of each procedure were also assessed. Finally, a survey of nursing staff was conducted to assess the professional impact of these techniques.

**Results:**

Tracheal aspiration had the lowest emissions (0.57 kgCO_2_e) and cost (€4), followed by BBS (2.82 kgCO_2_e, €24) and BAL (6.60 kgCO_2_e, €209). Nursing staff perceived BBS the most practical technique overall, and BAL the most technically demanding. In 2023, 341 procedures were performed in our ICU (73% BBS, 21% BAL, 6% TA), generating 1,181 kgCO2e and costing €20,835. Adopting TA exclusively in our ICU would reduce emissions by 84% and costs by 93%, whereas using BAL exclusively would increase emissions by 91% and costs by 242%.

**Conclusion:**

Bronchoalveolar lavage was associated with the highest carbon footprint and cost. These findings can help clinicians choose more sustainable methods for microbiological confirmation of VAP.

**Supplementary Information:**

The online version contains supplementary material available at 10.1186/s13613-025-01597-y.

## Introduction

Health-care systems account for approximately 5% of global greenhouse gas (GHG) emissions [1]. Intensive care units (ICUs) contribute significantly to this figure due to their high levels of technology and continuous operation [2–4]. The sourcing and production of drugs and medical devices are known to be among the main contributors to healthcare-related GHG emissions [1]. Subsequently, clinicians willing to mitigate ICUs environmental impact may prefer using procedures involving low-carbon-emission medical device, ensuring care remains safe and of high quality [5–8].

Ventilator-associated pneumonia (VAP) is the most frequent ICU-acquired infections and a major public health concern due to its associated morbidity and mortality [9]. Microbiological confirmation is strongly recommended [10–12]. Tracheal aspiration (TA), a simple and minimally invasive method, risks overdiagnosing pneumonia due to difficulty of distinguishing colonization from infection, potentially leading to antibiotic overuse [13, 14]. In contrast, lower respiratory tract sampling techniques such as bronchoalveolar lavage (BAL) and blind bronchial sampling (BBS) are more invasive procedures that require trained personnel. Both methods improve diagnostic accuracy [15]. However, evidence comparing invasive and non-invasive strategies remains contradictory: some studies reported reduced antibiotic use or even lower mortality with invasive approaches [13, 14], while others found no significant differences in patient outcomes [16–18]. To date, the optimal sampling method remains a matter of controversy. European guidelines recommend obtaining lower respiratory tract samples, with a preference for distal sampling (BAL and BBS), whereas American guidelines favor tracheal sampling with semiquantitative cultures [10, 11].

To the best of our knowledge, the environmental impact of these sample techniques has not been yet evaluated. The main objective of this study was to compare the carbon footprint of three respiratory sampling techniques for VAP. In addition, consistent with the three pillars of sustainability [19]—environmental, economic, and social—we also evaluated their costs and impacts on nursing staff experience.

## Methods

### Study design and setting

We conducted a prospective and observational study in one of the medical ICUs of La Pitié-Salpêtrière University Hospital in Paris, France. This hospital is part of Assistance Publique – Hôpitaux de Paris (AP-HP), which holds 38 public university hospitals mostly in the Grand Paris area.

The study focused on three standardized respiratory sampling techniques routinely performed in mechanically ventilated patients with suspected VAP (Figure S1, online supplement):

- Tracheal aspiration (TA) : blind aspiration of secretions through the endotracheal tube using a sterile suction catheter.
- Blind bronchial sampling (BBS): insertion of a protected specimen catheter through the endotracheal tube without direct visualization, obtaining distal airway secretions with reduced risk of contamination.
- Bronchoalveolar lavage (BAL) with single-use bronchoscope: performed under direct bronchoscopic guidance using a single-use flexible bronchoscope, allowing targeted sampling of distal airways.

We directly observed three procedures for each technique in routine care, without any intervention or modification of standard practice and without collecting any patient-identifiable data. We systematically documented every used item (drugs, medical devices, and consumables) employed and its packaging while timing the procedure.

### Ethical considerations

According to French law, the Ethics Committee of the French Intensive Care Society waived the need to submit the study, as no patient-identifiable data were collected.

### Study scope and functional unit

We performed a carbon footprint assessment in accordance with the International Organization for Standardization (ISO 14040/44) methodology [20]. As recommended, we defined the functional unit of this assessment as a single respiratory sampling procedure in a mechanically ventilated patient with suspected VAP, rather than all procedures performed in our ICU. This approach is more relevant for clinical practice and international comparisons. We then considered several carbon emission categories, including personal protective equipment (e.g., gloves, gowns, masks, eye protection), infection control supplies (e.g., antiseptic solution, sterile drapes), medication administration supplies (e.g., syringes, needles), sampling materials (e.g., suction probe, telescopic catheters, single use fiberscope, syringes, collection containers), sample handling and transportation (e.g., transport bags, specimen labels, biohazard packaging), staff-related emissions, biocleaning, laundry services and building energy use. Emissions from staff, biocleaning, and laundry services were calculated as a proportion of their total daily emissions, based on the time required for the procedure. Emissions from building energy use were estimated according to the fraction of the space occupied during the procedure.

We excluded from the system boundaries the laboratory analysis and waste processing of respiratory samples, as these steps have been considered comparable across the three techniques.

### Carbon footprint calculation method

#### GHG emissions

GHG emissions were expressed in kilograms of carbon dioxide equivalents (kgCO₂e), a standard metric that allows comparison of different GHGs relative to CO₂ using their 100-year Global Warming Potential. The carbon footprint of each system under study was calculated using the formula:

$$\:\text{G}\text{H}\text{G}\:\text{E}\text{m}\text{i}\text{s}\text{s}\text{i}\text{o}\text{n}\text{s}\hspace{0.17em}=\hspace{0.17em}\text{A}\text{c}\text{t}\text{i}\text{v}\text{i}\text{t}\text{y}\:\text{D}\text{a}\text{t}\text{a}\:\text{x}\:\text{E}\text{m}\text{i}\text{s}\text{s}\text{i}\text{o}\text{n}\:\text{F}\text{a}\text{c}\text{t}\text{o}\text{r}$$

Here, activity data represent the quantity of a specific activity (e.g., number of syringes used per procedure), while the emission factor (EF) indicates the carbon intensity associated with that activity.

#### Emission factors

A comprehensive database covering EF of all medical devices and related activities is currently unavailable. To accurately estimate EF, we applied a Life Cycle Assessment (LCA) methodology, which evaluates the environmental effects of a product across its entire life cycle—from raw material extraction to final disposal (“cradle-to-grave”).

The EF of each item in each emission category was estimated using a simplified process-based LCA methodology, compliant with ISO 14,040/44 standards. This analysis was conducted with the Carebone© tool developed by AP-HP in 2023 in France (https://bit.ly/carebone, see website for details) [21–26]. Unlike the economic input-output LCA method commonly used in similar studies, which assumes a proportional relationship between a product emissions and its cost—a relationship that is subject to large uncertainty —this methodology provides more precise EF for drugs and medical devices following a **“**cradle-to-grave” model, where each life cycle stage is parameterized with specific data for a given drug [27]. All EF used in this study are available within the Carebone© tool, which is open-source and freely accessible.

#### Uncertainty

In accordance with ISO 14,040 standards, uncertainty of carbon footprint calculation, which reflects the potential deviation between the quantified value and its true value, is reported. The total uncertainty for each respiratory sampling technique (TA, BBS, BAL) was calculated by the Carebone© tool.

#### Data analysis

Carbon footprint results are expressed in kgCO2e per procedure. Totals per procedure were rounded to one decimal, in order not to suggest a higher level of precision than supported by the data.

### Waste assessment

In addition to carbon footprint, we quantified the waste generated by each sampling technique. All components, including their packaging, were weighed. Results are expressed as weight (grams) of waste per procedure.

### Cost analysis

We estimated the total cost of each procedure by summing the acquisition costs of all medical devices and drugs—based on prices negotiated by AP-HP—and the staff-related costs. The latter were assessed using the Time-Driven Activity-Based Costing method, by calculating the time spent by each healthcare professional (nurses, assistant nurses, and physicians) and applying their hourly wages derived from the French public hospital salary scale, based on the specific durations observed for each technique [28, 29].

### Carbon footprint and cost over one year in our ICU

To quantify the overall carbon footprint and cost of these three sampling techniques over one year for our ICU, we measured the number of samples sent and analyzed by the microbiology department between January 1 and December 31, 2023. Because we could not isolate samples performed exclusively for the diagnosis of VAP, our analysis also included samples collected for the diagnosis of community-acquired or nosocomial pneumonia in patients who required mechanical ventilation upon admission, as well as BAL procedures performed in immunocompromised patients presenting with respiratory failure. Despite these limitations, the data enabled us to estimate the annual use of each diagnostic technique in our ICU and hence to calculate the corresponding carbon footprint and costs, and to model the potential impact if a single technique was uniformly applied throughout the year.

### Professional impact analysis

We conducted a survey among the nursing staff to gauge their preferences and experiences with the different techniques. It was developed specifically for this study, as no existing validated tool comparing these particular respiratory sampling techniques was available in the literature. The survey was designed to assess user-friendliness and ease of use for each method. It was validated through expert feedback from senior nursing staff and pre-tested on a sample of two nurses to ensure clarity and relevance of the questions. The survey was distributed via email and WhatsApp (Meta, USA) to the 41 nursing staff members in the ICU, with two reminders sent to maximize participation. The specific questions included in the survey are detailed in Table S1 in the Online Supplement.

## Results

### Materials used

Table 1 shows all different materials used in each sampling technique, divided into the five categories (personal protective equipment, infection control supplies, medication administration supplies, sampling materials and sample handling and transportation), with corresponding carbon emission and cost. Besides “personal protective equipment” and “sample handling and transportation”, each sampling technique uses specific materials.

Bronchoalveolar lavage emitted 2.3 times more CO_2_e compared to BBS and 11.3 times more than TA, while BBS emitted 4.9 times more CO_2_e than TA.

**Table 1:** Carbon footprint and cost of materials used for each sampling technique, as well as usage frequency

	Carbon emission, kgCO_2_e	Cost, €	Number of units used for one tracheal aspiration	Number of units used for one blind bronchial sampling	Number of units used for one broncho alveolar lavage
***Personal protective equipment***					
Protective apron	0.12	0.03	1	2	3
Surgical cap	0.04	0.30	1	2	3
Protective goggles^1^	0.36	1.49	1	2	1
Pair of non-sterile gloves	0.07	0.04	1	1	3
Pair of sterile gloves	0.10	0.32		1	
***Infection control supplies***					
Single-use bottle of antiseptic solution	0.33	1.84		1	1
Pack of sterile non-woven compresses	0.09	0.04		2	2
Sterile surgical drape	0.43	0.24		1	
***Medication administration supplies***					
Atracurium solution for injection	0.41	1.41			1
Transfer needle	0.02	0.03			1
10 ml syringe	0.03	0.04			1
***Sampling materials***					
2 ml syringe	0.02	0.02		1	
20 ml syringe	0.07	0.11		1	
50 ml syringe	0.15	0.16			2
Sodium chloride 0.9%, 5 ml vial	0.03	0.03		1	
Sodium chloride 0.9% / 500 ml vial	0.56	0.80			1
Mucus extractor 80 ml	0.02	1.18	1		
Screw cap jars for bacteriological sampling 40 ml	0.06	0.57		1	1
Double male-female connector	0.02	0.02	2		
Lubricating gel 2 g	0.09	0.42			1
PE/PVC extension pipe 2.5 mm 25 cm, 3-way tap	0.04	0.30			1
Single-use protective sheet	0.21	0.16			1
Tracheo-bronchial suction probe 50 cm	0.05	0.07		1	
Sterile scissors	0.26	0.27		2	
Bronchial sampling catheter Aspisafe 2 (Vygon, United-Kingdom)	0.03	13.11		1	
Single use fiberscope –aScope™ 4 Broncho Regular (Ambu, Denmark)	2.24	180			1
***Sample handling and transportation***					
Double bag for biological samples sending	0.02	0.03	1	1	1
Patient label		0.04	2	2	2
Service label		0.04	1	1	1
Bacteriological test request form		0.08	1	1	1

kgCO_2_e: kilograms of carbon dioxide equivalents; PE/PVC: polyethylene/ polyvinyl chloride

^1^ Multiples uses, not counted in final result

### Carbon footprint analysis

**Fig. 1:**
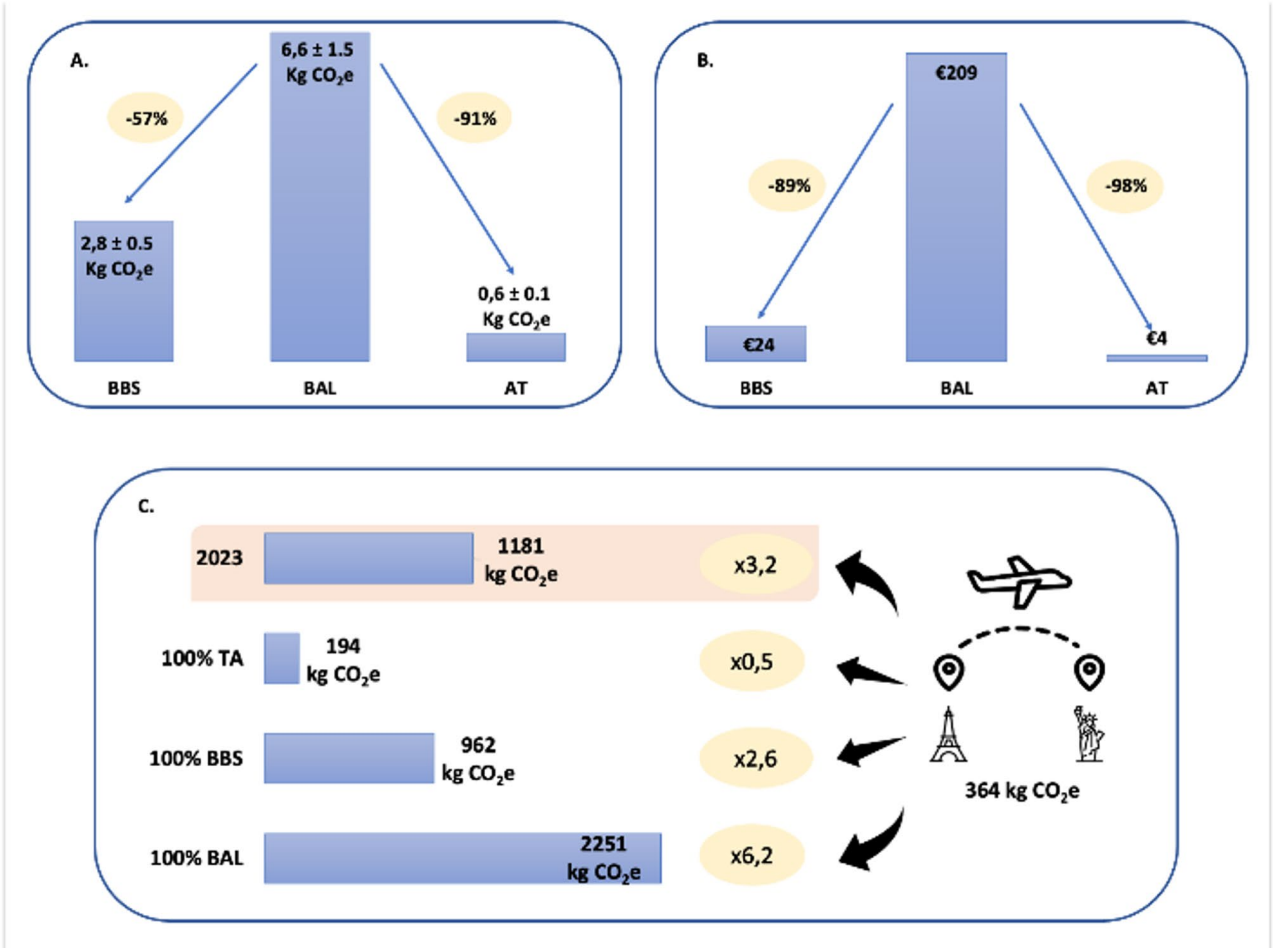
Carbon footprint and cost of three respiratory sampling techniques. (A) Carbon footprint (kgCO₂e) of each sampling technique. (B) Cost (€) of each sampling technique. (C) Total annual carbon footprint in our ICU (2023) based on actual use of each technique, with hypothetical projections if only one method were used exclusively. The projected emissions are expressed in number of one-way Paris–New York flights (1 flight = 364 kgCO₂e). kgCO_2_e, kilograms of carbon dioxide equivalents; TA, tracheal aspiration; BBS, blinded bronchial sampling; BAL, bronchoalveolar lavage

The carbon footprint for each respiratory sampling was 0.6 ± 0.1 kgCO_2_e for TA, 2.8 ± 0.5 kgCO_2_e for BBS and 6.6 ± 1.5 kgCO_2_e for BAL (Table 2). These differences are illustrated graphically in Fig. 1. For BAL, the single-use bronchoscope alone was estimated to be responsible for 2.2 ± 0.6 kgCO_2_e, and the preparation of neuromuscular blockers (drug and material use, plus 2 min of nurse time) for 0.9 ± 0.4 kgCO_2_e (Table 1).

**Table 2:** Staff time allocation, carbon footprint and cost for each respiratory sampling technique

	Tracheal aspiration	Blind bronchial sampling	Bronchoalveolar lavage
***Staff time allocation***			
Nurse, *min*	8	12	32^1^
Nursing assistant, *min*	0	12	20^1^
Physician, *min*	0	0	20^1^
***Carbon footprint by category***			
Staff-related emissions, *kgCO*_*2*_*e*	0.11	0.34	1.02
Personal protective equipment, *kgCO*_*2*_*e*	0.23	0.49	0.69
Infection control supplies, *kgCO*_*2*_*e*	0	0.94	0.51
Medication administration supplies, *kgCO*_*2*_*e*	0	0	0.46
Sampling materials, *kgCO*_*2*_*e*	0.06	0.78	3.50
Sample handling and transportation, *kgCO*_*2*_*e*	0.02	0.02	0.02
Biocleaning, *kgCO*_*2*_*e*	0.10	0.13	0.13
Laundry services, *kgCO*_*2*_*e*	0.01	0.04	0.11
Building energy use, *kgCO*_*2*_*e*	0.04	0.08	0.16
Estimated relative margin of error, %	22	19	25^2^
**Total carbon footprint estimates per procedure**, ***kgCO***_***2***_***e***^***3***^	**0.6 ± 0.1**	**2.8 ± 0.5**	**6.6 ± 1.5**
**Waste generated by each technique**, ***g/procedure***	165	295	1125
***Cost by category*** ^**4**^			
Staff-related cost, *€*	2.19	5.92	21.84
Personal protective equipment, *kgCO*_*2*_*e*	0.37	1.02	1.11
Infection control supplies, *kgCO*_*2*_*e*	0	2.16	1.92
Medication administration supplies, *kgCO*_*2*_*e*	0	0	1.48
Sampling materials, *kgCO*_*2*_*e*	1.22	14.45	182.57
Sample handling and transportation, *kgCO*_*2*_*e*	0.23	0.23	0.23
**Total cost estimates per procedure**, ***€*** ^**3**^	**4.0**	**23.8**	**209.2**

kgCO_2_e, kilograms of carbon dioxide equivalents

^1^ 2 min for neuromuscular blockade administration

^2^ 42% uncertainty for neuromuscular blockade preparation

^3^ results are rounded to one decimal, in order not to suggest a higher level of precision than supported by the data

^4^ Biocleaning, laundry services, building energy use, and printed materials were excluded from the cost analysis due to their negligible cost contribution per procedure

### Waste assessment

Regarding the waste generated by each technique, which is mostly plastic, TA generated the lowest amount of waste (165 g/procedure), followed by BBS (295 g/procedure), while BAL with single-use bronchoscopes produced the highest amount (1125 g/procedure). These data are summarized in Table 2.

### Cost analysis

The cost analysis revealed differences between the techniques (Table 2) which are visually summarized in Fig. 1. The cost per procedure was €4 for TA, €24 for BBS and €209 for BAL. Bronchoalveolar lavage was 9 times more expensive than BBS and 52 times more expensive than TA, while BBS was 6 times more expensive than TA.

### Carbon footprint and cost over one year in our ICU

In 2023, a total of 341 respiratory sampling procedures were performed in our ICU, with 73% (*n* = 248) being BBS, 21% (*n* = 71) BAL, and 6% (*n* = 22) TA. The total carbon footprint for these procedures was 1181 kgCO_2_e, with BBS accounting for 699 kgCO_2_e, BAL for 469 kgCO_2_e, and TA for 13 kgCO_2_e (Fig. 1). The total cost for these procedures was €20,835, with BBS costing €5,897, BAL costing €14,850, and TA costing €88.

We projected the carbon emissions and costs if our ICU adopted a single-technique strategy for all procedures. For 100% BBS it would represent 962 kgCO_2_e and €8,109. For 100% BAL it would represent 2,251 kgCO_2_e and €71,320. For 100% TA it would represent 194 kgCO_2_e and €1,367. Switching entirely to TA would result in an 84% reduction in carbon footprint and a 93% reduction in costs compared to the current distribution. If our ICU adopted BBS exclusively, the carbon footprint would decrease by 19% and costs by 61%. Conversely, adopting BAL exclusively would increase the carbon footprint by 91% and costs by 242%.

### Professional impact analysis

The survey had a response rate of 73% (30/41), with respondents being predominantly female (73%) and mostly under the age of 30 (66%). 40% had less than two years’ experience in the profession, while 53% reported between two and five years of experience.

When asked about the technical difficulty of the procedures, 87% (*n* = 26) considered TA to be the least challenging, whereas 83% (*n* = 25) identified BAL as the most technically demanding. Tracheal aspiration was also ranked as the fastest procedure by 93% (*n* = 28), while BAL was unanimously reported as the slowest (*n* = 30).

Regarding first-attempt success (i.e., no need to repeat sampling), 47% (*n* = 14) of respondents rated BAL as the most effective technique, while 57% (*n* = 17) identified TA as the least effective. The need to repeat sampling is not based on predefined criteria but on the operator’s judgment when the initial sample was considered of insufficient quality. Finally, when asked which sampling technique they found most practical and convenient to perform in their daily workflow, 63% of nurses favored BBS, 30% preferred TA, and only 7% selected BAL.

## Discussion

In this study, we evaluated the carbon footprint, cost and professional impact of three different respiratory sampling techniques used to diagnose VAP. Our findings revealed that both BBS and TA have a lower carbon footprint and associated costs compared to BAL with single-use bronchoscope. To our knowledge, this is the first study to compare the carbon footprint of these different sampling techniques, and these findings provide valuable insights for clinical teams when selecting a sampling method. Beyond diagnostic performance, the environmental impact of each technique should also be considered—especially when assessing their cumulative footprint over a full year of activity in a single ICU. In 2023, respiratory sampling procedures in our unit accounted for a total carbon footprint equivalent to 3.2 round-trip flights from Paris to New York (1 flight = 364 kgCO₂e [30]) (Fig. 1). If a single-technique approach were adopted throughout the year, the projected emissions would vary significantly: 2 flights for 100% BBS, 6.2 flights for 100% BAL and 0.5 flights for 100% TA. These variations underscore how the choice of technique, while clinically driven, can also represent a lever for reducing the carbon footprint of intensive care medicine without compromising care.

While environmental impact is a critical dimension, it cannot be considered in isolation from clinical performance. Although TA had the lowest carbon footprint per procedure in our analysis, this apparent advantage must be interpreted with caution. The risk of false positives may contribute to unnecessary antibiotic prescriptions [13], which carry a substantial environmental impact themselves [31], potentially offsetting the initial ecological benefit of TA. Beyond their immediate carbon footprint, the inappropriate use of antibiotics also contributes to the emergence of resistant bacteria [32]. BBS results in a moderate carbon footprint and cost, is relatively simple to perform, and provides good diagnostic specificity, supporting targeted antibiotic use [33]. BAL performed with single-use bronchoscopes is the most resource-intensive and costly approach, requiring more staff time and being associated with non-negligible adverse events [34]; however, it remains highly specific and contributes to optimized antibiotic stewardship [13, 14]. In light of these considerations, BBS may represent the most pragmatic balance between diagnostic efficacy and environmental responsibility. In our study, BBS was also identified as the preferred technique by most nurses in terms of integration into their daily workflow. This preference may reflect their greater familiarity with the procedure and the relative ease of its implementation in our ICU. While this finding is contextual and may vary between centers, it highlights the importance of aligning clinical practices not only with diagnostic performance and environmental considerations, but also with the realities of frontline staff. This approach is consistent with the broader principles of sustainable healthcare, which aim to deliver high-quality, safe, and clinically relevant care while minimizing environmental impact.

Beyond carbon footprint, our analysis also underscores the environmental burden of plastic waste. While TA and BBS produced relatively modest amounts, BAL with single-use bronchoscopes generated substantially higher quantities, largely due to the disposable bronchoscope and its packaging. Plastic waste poses an additional challenge for critical care sustainability, contributing both to resource consumption and waste management.

One of the main strengths of our study is its pragmatic approach, focusing on routine ICU practices with materials and procedures commonly used in many settings. This makes our findings likely transposable to other ICUs, both in France and internationally, as the processes involved in performing these sampling techniques are relatively standardized despite the fact that this is a single-center design. This contrasts with previous studies that compared single-use and reusable bronchoscopes [35, 36]. In such cases, the carbon footprint of reusable bronchoscopes can vary greatly depending on the energy mix of the country studied, with sterilization processes being far more carbon-intensive in countries reliant on fossil fuels. By focusing instead on routine respiratory sampling techniques with materials commonly used across ICUs, our study provides results that are more broadly relevant and transferable to different healthcare systems.

Our study has several limitations. First, the study only evaluates single-use bronchoscopes, limiting its applicability to centers that use this technology. Previous studies have shown that, over multiple uses, reusable bronchoscopes result in a lower carbon footprint than single-use devices [35, 36]. However, in our analysis, the single-use bronchoscope accounted for 2.2 kgCO2e out of the total 6.6 kgCO2e for BAL. Even if this contribution were excluded—which would underestimate the impact since a reusable bronchoscope also entails emissions—the remaining footprint (≈ 4.4 kgCO2e) would still exceed that of the other sampling techniques, even when accounting for uncertainty.

Second, our estimates carry inherent uncertainty (19–25% across techniques) as reported by the Carebone© tool, which provides a complete life cycle assessment of drugs and medical devices but excludes research and development. We did not include the carbon footprint of laboratory analyses or the downstream waste processing of respiratory samples, which may slightly underestimate the absolute footprint. Nevertheless, these limitations are unlikely to alter the relative differences or the overall ranking between TA, BBS, and BAL.

Third, our study includes all samples analyzed by the microbiology laboratory in 2023, encompassing not only suspected VAP but also community-acquired or nosocomial pneumonias requiring mechanical ventilation on admission, respiratory failure in immunocompromised patients, in whom BAL remains essential. This approach necessarily overestimated the actual number of VAP-related procedures. As such, a complete shift to 100% TA or BBS is neither realistic nor desirable. Nevertheless, BAL can provide a high level of input for clinical decision-making, but it is not without risk, with adverse events occurring in approximately 14% of cases, often related to the operator’s experience [34].

Fourth, technical procedures may vary between centers; for example, neuromuscular blockade during BAL is commonly used in our ICU but may not be standard practice elsewhere.

Fifth, from the nurses’ perspective, both TA and BBS may occasionally require repeated attempts to obtain a usable sample. While this could increase the associated carbon footprint and cost, in practice, only the sampling device is reused—not all materials—limiting the additional impact. Although we could not precisely quantify the frequency of repeated attempts, it is likely that even with occasional repetitions, the overall environmental and economic burden of TA and BBS remains substantially lower than that of BAL.

## Conclusion

BAL is associated with higher environmental and economic costs compared to other respiratory sampling techniques. Considering the significant carbon footprint of ICUs, these findings can help guide clinicians towards more sustainable and cost-effective diagnostic strategies. Further studies should specifically address the environmental impact of antimicrobial stewardship, infection diagnostic strategies, and device reprocessing in the ICU, in order to identify actionable targets for a more sustainable model of critical care.

## Supplementary Information

Below is the link to the electronic supplementary material.

Supplementary Material 1

## Data Availability

The datasets used and/or analysed during the current study are available from the corresponding author on reasonable request.
