# Supplementary material for: Choosing wisely: comparing the carbon footprint of three respiratory sampling techniques for ventilator-associated pneumonia
Source: Ann Intensive Care. 2025 Dec 8;15:191. doi: 10.1186/s13613-025-01597-y (PMC12686224; doi:10.1186/s13613-025-01597-y)
Supplement: Supplementary file 1 — Supplementary Material 1 [file 13613_2025_1597_MOESM1_ESM.docx]

**Choosing wisely: comparing the carbon footprint of three respiratory sampling techniques for ventilator-associated pneumonia**

**Online supplement**

**Table S1. Survey on respiratory sampling techniques among ICU nurses**

| **Question** |
| --- |
| *Are you : a woman / a man / gender neutral* |
| *What is your age group?* |
| *For how long have you worked in the intensive care unit?* |
| *Rank these three methods (tracheal aspiration, blind bronchial sampling and bronchoalveolar lavage) according to their technical ease of execution (considering material preparation, performing the procedure, and sample submission).* |
| *Rank these three methods from the quickest to the slowest (considering material preparation, performing the procedure, and sample submission).* |
| *Rank these three methods according to their efficiency, based on your experience (successful sampling on the first attempt, no need to repeat the sample).* |
| *Ultimately, which technique do you find most practical and convenient to perform in your daily workflow?* |

**Supplemental figure legend**

**Figure S1. Description and comparison of the three sampling techniques**

Schematic comparison of tracheal aspirate, blind bronchial sampling, and bronchoalveolar lavage. The figure highlights differences in sampling site (proximal vs. distal), guidance (blinded vs. fiberoptic-guided), invasiveness, complexity, procedure duration, number of caregivers required, and need for sedation.

Image adapted from [https://bioart.niaid.nih.gov/](https://bioart.niaid.nih.gov/?utm_source=chatgpt.com) which are public domain and free to use.
